# Altered Functional Connectivity of the Basal Nucleus of Meynert in Subjective Cognitive Impairment, Early Mild Cognitive Impairment, and Late Mild Cognitive Impairment

**DOI:** 10.3389/fnagi.2021.671351

**Published:** 2021-06-25

**Authors:** Wenwen Xu, Jiang Rao, Yu Song, Shanshan Chen, Chen Xue, Guanjie Hu, Xingjian Lin, Jiu Chen

**Affiliations:** ^1^Department of Neurology, The Affiliated Brain Hospital of Nanjing Medical University, Nanjing, China; ^2^Department of Rehabilitation, The Affiliated Brain Hospital of Nanjing Medical University, Nanjing, China; ^3^Department of Radiology, The Affiliated Brain Hospital of Nanjing Medical University, Nanjing, China; ^4^Institute of Neuropsychiatry, The Affiliated Brain Hospital of Nanjing Medical University, Nanjing, China; ^5^Institute of Brain Functional Imaging, Nanjing Medical University, Nanjing, China

**Keywords:** early mild cognitive impairment, late mild cognitive impairment, subjective cognitive impairment, basal nucleus of Meynert, functional connectivity, basal forebrain

## Abstract

**Background:** The spectrum of early Alzheimer's disease (AD) is thought to include subjective cognitive impairment, early mild cognitive impairment (eMCI), and late mild cognitive impairment (lMCI). Choline dysfunction affects the early progression of AD, in which the basal nucleus of Meynert (BNM) is primarily responsible for cortical cholinergic innervation. The aims of this study were to determine the abnormal patterns of BNM-functional connectivity (BNM-FC) in the preclinical AD spectrum (SCD, eMCI, and lMCI) and further explore the relationships between these alterations and neuropsychological measures.

**Methods:** Resting-state functional magnetic resonance imaging (rs-fMRI) was used to investigate FC based on a seed mask (BNM mask) in 28 healthy controls (HC), 30 SCD, 24 eMCI, and 25 lMCI patients. Furthermore, the relationship between altered FC and neurocognitive performance was examined by a correlation analysis. The receiver operating characteristic (ROC) curve of abnormal BNM-FC was used to specifically determine the classification ability to differentiate the early AD disease spectrum relative to HC (SCD and HC, eMCI and HC, lMCI and HC) and pairs of groups in the AD disease spectrum (eMCI and SCD, lMCI and SCD, eMCI and lMCI).

**Results:** Compared with HC, SCD patients showed increased FC in the bilateral SMA and decreased FC in the bilateral cerebellum and middle frontal gyrus (MFG), eMCI patients showed significantly decreased FC in the bilateral precuneus, and lMCI individuals showed decreased FC in the right lingual gyrus. Compared with the SCD group, the eMCI group showed decreased FC in the right superior frontal gyrus (SFG), while the lMCI group showed decreased FC in the left middle temporal gyrus (MTG). Compared with the eMCI group, the lMCI group showed decreased FC in the right hippocampus. Interestingly, abnormal FC was associated with certain cognitive domains and functions including episodic memory, executive function, information processing speed, and visuospatial function in the disease groups. BNM-FC of SFG in distinguishing eMCI from SCD; BNM-FC of MTG in distinguishing lMCI from SCD; BNM-FC of the MTG, hippocampus, and cerebellum in distinguishing SCD from HC; and BNM-FC of the hippocampus and MFG in distinguishing eMCI from lMCI have high sensitivity and specificity.

**Conclusions:** The abnormal BNM-FC patterns can characterize the early disease spectrum of AD (SCD, eMCI, and lMCI) and are closely related to the cognitive domains. These new and reliable findings will provide a new perspective in identifying the early disease spectrum of AD and further strengthen the role of cholinergic theory in AD.

## Introduction

Subjective cognitive impairment (SCD), characterized by a condition where self-aware cognitive function is diminished, is reported to be a very early stage of the preclinical AD spectrum (Lautenschlager et al., 2019). Numerous credible studies proved that healthy people with SCD have a higher risk of conversion to amnestic mild cognitive impairment (aMCI) and dementia, the progression rate of which is twice than healthy people without SCD per year (Lautenschlager et al., 2019). As a subtype of MCI, aMCI, defined by exact existing memory disorders, is considered to be the distinctive transitional stage converting to AD (Csukly et al., 2016). Given the more refined early diagnosis and discrimination, aMCI is categorized into early MCI (eMCI) and late MCI (lMCI) according to the severity of the memory impairment assessed by neuropsychological examinations (Edmonds et al., 2019). Neuroimaging has been recognized as a good indicator of the pathologic progression of AD (Chen S. et al., 2020). As a result, the brain imaging characteristics of the preclinical stages of the AD spectrum including SCD, eMCI, and lMCI are worth further studying.

To explain the pathogenesis of AD with a hypothesis is limited and difficult (Barage and Sonawane, 2015). The pathogenesis of AD is very complex and has not yet been fully elucidated (Barage and Sonawane, 2015). The known pathogenesis is associated with cholinergic deficiency, gene mutation, oxidative stress, free radical injury, and inflammatory immune injury (Chen, 2018). At present, the mainstream view early recognized internationally is the cholinergic hypothesis, that is, neurocholinergic neuronal degeneration or disruption of the cholinergic system in the brain (Hampel et al., 2019). Acetylcholine (ACh), a neurotransmitter linked with learning, memory, and neuromodulation, is provided from the basal forebrain (BF) to the neocortex, hippocampus, and amygdala (Hampel et al., 2019). In addition, four overlapping cell groups (Ch1–Ch4) are contained in the BF system (Liu et al., 2015). Supported by relevant literature, a broad band of cell clusters named the basal nucleus of Meynert (BNM) is located in the BF (Gratwicke et al., 2020). Specifically, BNM is an anatomical structure corresponding to the Ch4 group which is the largest group of four groups. In the very early course of the neurodegenerative disease, previous studies have confirmed that downregulation of cholinergic markers exactly occurs (Fotiou et al., 2015). Thus, as the key hub for cholinergic energy, the study of BNM shows the great significance of cholinergic deficiency which might represent an etiological marker of cognitive impairment in the early stage of AD.

Increasing neuroimaging techniques such as resting-state functional magnetic resonance imaging (fMRI) are being applied to assess brain alterations in SCD, MCI, and AD patients to further understand the pathogenesis and progression (Fotiou et al., 2015). The disruption of neural circuit connectivities within the default mode network (DMN), executive control network (ECN), and salience network has been deeply demonstrated (Xu et al., 2020). Based on the stereotaxic map of the BNM constructed by Zaborszky et al., two latest studies show that functional disconnection exists in the BNM with insula/claustrum, leading to cognitive decline (Li et al., 2014). A study assessed volume reductions of the cholinergic basal forebrain nuclei in SCD subjects on 3D-T1-weighted MR images based on a postmortem-derived atlas (Scheef et al., 2019). Decreased volume of BNM in AD has been further reported in AD and MCI patients. Postmortem studies also have shown evidence that neuronal loss in the BNM are selectively vulnerable to degeneration in AD (Grothe et al., 2012). Dubois et al. (2016) conformed even if the criteria for cognitive impairment are not met that the diagnosis of preclinical AD can be made as long as the presence of biomarkers of amyloid beta and tau is detected by pathology. In other words, AD-specific pathological changes such as neuroimaging markers and biochemical pathological markers occur in the human brain decades before clinical symptoms appear (Tan et al., 2014). Moreover, the degree of BNM atrophy shows a linear correlation with amyloid-beta burden from the *in vivo* MRI outcomings, which is the other important pathologic mechanism of AD (Grothe et al., 2013). Therefore, as an important anatomical structure in cholinergic theory, whether in terms of structure or function, the alteration of BNM in early AD spectrum in detail deserves attention.

So far, although evidence can only support the differences in the FC of the BNM in AD and MCI, there has been no study of BNM in the detailed early AD spectrum (Li et al., 2017). Hence, it is important to determine alterations in BNM-functional connectivity (BNM-FC) in individuals in the early disease spectrum of AD and to explore the relationships between BNM-FC and neuropsychological scores. We hypothesize that (1) abnormal BNM-FC among the early AD spectrum can be detected and continuous disruption of brain functional networks during disease progression can be explored, (2) these abnormal FCs are linked with impairments in different cognitive domains, and (3) the ability of these abnormal brain regions to classify the early AD disease spectrum is highly specific and sensitive.

## Method

### Participants

A total of 116 elderly individuals participated in our study, including 30 HC, 30 SCD, 28 eMCI, and 28 lMCI. All of the participant data came from our in-home database: the Nanjing Brain Hospital-Alzheimer's Disease Spectrum Neuroimaging Project (NBH-ADsnp) (Nanjing, China). Supplementary Materials summarized specific information about NBH-ADsnp in detail. Seven of them were ruled out because of no MRI data and two of them were excluded due to the effect of head motion (cumulative translation or rotation >3.0 mm or 3.0°) (Xue et al., 2020). At last, 107 individuals were included (28 HC, 30 SCD, 24 eMCI, and 25 lMCI) in the present study. Relevant exclusion criteria and inclusion criteria were documented in the Supplementary Materials.

### Neurocognitive Assessments

All participants underwent comprehensive and standard neurocognitive assessments to evaluate their cognitive function, including general cognitive functions, episodic memory, executive function, information processing speed, and visual spatial domains (Gu et al., 2017; Chen J. et al., 2020). Comprehensive division and evaluation details were summarized in the Supplementary Materials.

### MRI Data Acquisition

Detailed MRI data acquisition parameters involved in the NBH-ADsnp database were listed in Supplementary Materials.

### MRI Data Preprocessing

On the basis of Statistical Parametric Mapping (SPM8), fMRI data were preprocessed by MATLAB2013b and Data Processing and Analysis for Brain Imaging (DPABI). In summary, the concrete steps of image preprocessing were provided in the Supplementary Materials. Overall, slice-timing and head motion correction, realignment, nuisance covariate regression, normalization, smoothing, and filtering were a series of steps in the process.

### Functional Connectivity Analysis

A seed-based FC analysis was performed to explore the alternation of AD early disease spectrum (Li et al., 2017). The FC method based on seed voxel analysis can identify the brain regions that are related to the function of the seed voxel selected initially (Bell et al., 2019). If the brain regions and seed regions show a high degree of time-domain consistency, it can be considered that these brain regions and seed brain regions together form a network related to a certain brain function (Joel et al., 2011). The identification and fabrication of seed points for BNM were summarized in the Supplementary Materials.

### Statistical Analyses

SPSS 19 was used to analyze clinical data and neuropsychological measures. The analysis of variance (ANOVA), the multimodal general linear model (GLM), and the chi-square test were conducted to compare the demographic and neurocognitive data among groups, including the HC, SCD, eMCI, and lMCI. The Bonferroni correction was used for *post-hoc* comparisons. The *p*-value was set as <0.05 for significant differences.

One-way ANOVA analysis was performed to determine the differential brain regions among the four groups with the control of the influence of age, gender, and education level. Then, the two-sample *t*-test was used for comparing differential BNM-FC in any two of the four groups based on the mask resulted from ANOVA analyses after controlling for the effects of age, gender, and level of education. Results within-group were thresholded at voxel level *p* < 0.05 [Gaussian random field (GRF) corrected] and cluster size >50 voxels. The FC values of the altered regions were extracted with DPABI.

Notably, four cognitive domains were divided from the neuropsychological tests (Supplementary Materials). These original psychometric scores were converted to standardized *Z*-values. These initial psychological scores were converted to standardized *Z*-values, which were then added together to obtain the cognitive domain average (Gao et al., 2018; Chen et al., 2019). A correlation analysis was conducted between altered BNM-FC and cognitive domains (Bonferroni corrected, *p* < 0.05).

In addition, the ability of altered BNM-FC in specific brain regions to differentiate and classify the spectrum of early AD diseases was realized by ROC curve (Pei et al., 2018). First, the FC value and group number were analyzed by binary regression to obtain the prediction probability. Then, the ROC curve of the prediction probability and the group was analyzed. The FC values of multiple brain regions were synthesized during binary regression analysis to obtain a new prediction probability.

## Results

### Demographic and Neurocognitive Characteristics

The demographic and neurocognitive information of all participants, including 28 HC (mean age 62.43 ± 13.39), 30 SCD (mean age 66.23 ± 14.01), 24 eMCI (mean age 62.96 ± 13.97), and 25 lMCI (mean age 66.28 ± 15.06) individuals, can be found in Table 1. As expected, our results showed significant differences in cognitive performance between pairs of the comparison groups. Compared with HC, both the SCD group and the eMCI group showed lower MDRS-2 and MoCA and showed higher SCD-Q scores. In addition, the eMCI group also showed lower EM score and IPS score than the HC group and the SCD group. Compared with the eMCI group, the lMCI group exhibited significantly lower MDRS-2, MoCA, EM score, and VF score. The lMCI group also showed lower EF score and SCD-Q score than the SCD group and higher SCD-Q score than the HC group (all *p* < 0.05).

**Table 1:** Demographics and clinical measures of HC and patients with SCD, eMCI, and lMCI.

	**HC**	**SCD**	**eMCI**	**lMCI**	***F*-values (χ^**2**^)**	***p*-values**
	***N* = 28**	***N* = 30**	***N* = 24**	***N* = 25**		
Age (years)	62.43 ± 13.39	66.23 ± 14.01	62.96 ± 13.97	66.28 ± 15.06	1.918	0.131
Gender (m/f)	15/13	7/23	7/17	7/15	6.523	0.089
Education (years)	12.64 ± 3.20	12.05 ± 2.77^*^	11.46 ± 3.35^*^	11.16 ± 3.44^&^	1.665	0.179
MMSE	28.57 ± 5.09	27.97 ± 4.80	27.33 ± 5.41	26.76 ± 5.13	9.977	<0.001
MDRS-2	141.89 ± 26.03	139.60 ± 25.05^*^	137.42 ± 27.49^*^	135.56 ± 26.75^&^	11.037	<0.001
MoCA	26.08 ± 5.25	24.50 ± 4.57^*^	23.17 ± 5.04^*^	21.72 ± 4.82^&^	13.464	<0.001
SCD-Q	3.31 ± 1.39	6.52 ± 1.28^*^	4.66 ± 2.07^*^	5.62 ± 1.80^*^^#^	21.861	<0.001
**Composite** ***Z*****-scores of each cognitive**						
EM	0.00 ± 0.69	−0.01 ± 0.54	−0.17 ± 0.60^*^^#^	−0.76 ± 0.77^&^	13.230	<0.001
IPS	0.00 ± 0.61	0.05 ± 0.44	−0.17 ± 0.59^#^	0.29 ± 0.67	3.501	0.018
EF	0.00 ± 0.55	0.24 ± 0.74	−0.23 ± 0.65	0.03 ± 0.61^#^	4.269	0.007
VF	0.00 ± 0.78	0.28 ± 0.64	−0.14 ± 1.04	−0.35 ± 0.92^&^	3.270	0.024

*Numbers are given as means (standard deviation, SD) unless stated otherwise. Scores reflect the number of correct items unless stated otherwise. Significant group differences were found at p <0.05 (ANOVA test and multivariable general linear model), Bonferroni corrected. Between-group comparisons were further utilized to reveal specific alterations among matched groups (*

* *compared with HC*,

# *compared with SCD*,

& *compared with eMCI)*.

*MMSE, Mini-Mental State Exam; MDRS-2, Mattis Dementia Rating Scale-2; MoCA, the Montreal Cognitive Assessment test; SCD-Q, Subjective Cognitive Decline Questionnaire; HC, healthy controls; SCD, subjective cognitive decline; eMCI, early mild cognitive impairment; lMCI, late mild cognitive impairment; EM, episodic memory; EF, executive function; VF, visuospatial function; IPS, information processing speed*.

**Figure 1:**
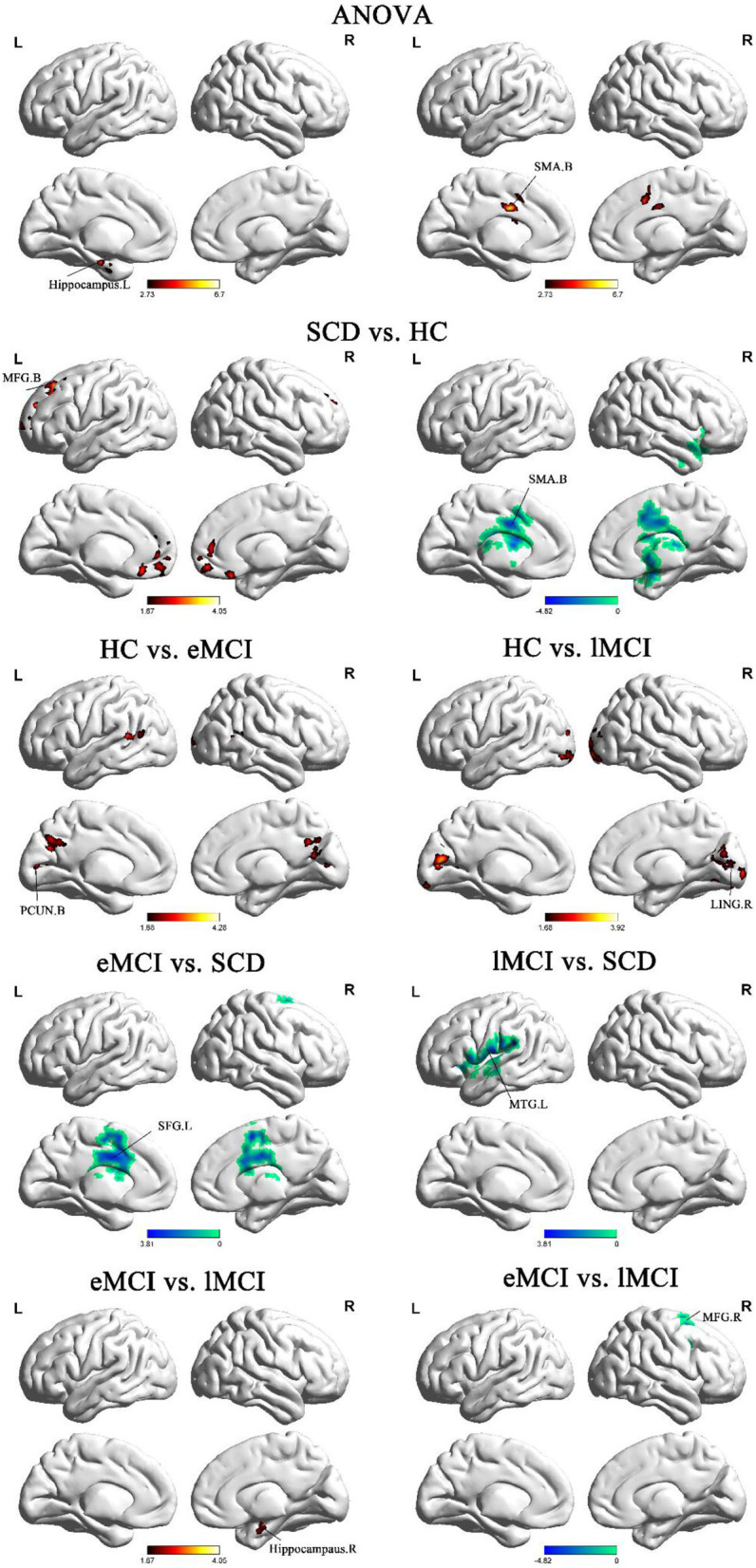
Brain regions exhibiting significant differences in BNM-FC based on analysis of variance (ANOVA) analysis and two-sample *t*-tests. Age, gender, and years of education were used as covariates for all these results. GRF corrected, cluster size ≥50 mm^3^, *p* < 0.05. eMCI, early mild cognitive impairment; lMCI, late mild cognitive impairment; SCD, subjective cognitive decline; HC, healthy controls; SMA, supplementary motor area; PCUN, precuneus; SFG, superior frontal gyrus; MFG, middle frontal gyrus; MTG, middle temporal gyrus; LING, lingual gyrus; L, left hemisphere; R, right hemisphere; B, bilateral hemisphere.

The ANOVA analysis showed three significantly altered brain regions among the four groups, including the left hippocampus, bilateral cerebellum, and bilateral supplementary motor area (SMA). Compared with HC, eMCI patients showed significantly decreased BNM-FC in the bilateral precuneus (PCUN), lMCI individuals showed decreased BNM-FC in the right lingual gyrus (LING), and SCD patients showed increased BNM-FC in the bilateral SMA and decreased BNM-FC in the bilateral cerebellum and middle frontal gyrus (MFG). Compared with the SCD group, the eMCI group showed decreased BNM-FC in the right superior frontal gyrus (SFG), while the lMCI group showed decreased BNM-FC in the left middle temporal gyrus (MTG). Compared with the eMCI group, the lMCI group showed decreased BNM-FC in the right hippocampus. All the results were based on controlling for age, gender, and level of education (GRF corrected, cluster size ≥50 mm^3^, *p* < 0.05) (Figure 1 and Table 2).

**Table 2:** Regions of BNM-FC based on analysis of variance (ANOVA) analysis and two-sample *t*-tests.

**Region**	**MNI**			***F*/*t***	**Cluster number**
	***x***	***Y***	***z***		
**ANOVA**					
L hippocampus	−21	3	−12	6.7027	61
B cerebellum	−12	−60	−54	8.0209	140
B supplementary motor area	−9	−12	33	6.9442	195
**eMCI < HC**					
B precuneus	−33	−75	0	4.286	1,098
**lMCI < HC**					
R lingual gyrus	18	−84	3	3.9196	1,521
**SCD>HC**					
B supplementary motor area	−9	−12	33	−4.8206	1,032
**SCD < HC**					
B middle frontal gyrus	−18	33	48	3.8688	1,177
B cerebellum	−12	−54	−51	4.0489	451
**eMCI>lMCI**					
R hippocampus	27	−6	−24	3.6856	151
**eMCI < lMCI**					
R middle frontal gyrus	30	9	54	−3.3575	118
**eMCI < SCD**					
R superior frontal gyrus	−9	−12	36	−3.8082	638
**lMCI < SCD**					
L middle temporal gyrus	−66	−21	24	−4.5037	306

*The x, y, z coordinates are the primary peak locations in the MNI space. Cluster size >50 mm^3^ in ANOVA analysis, p <0.05; cluster size >50 mm^3^ in the two-sample t-test, p < 0.05, GRF corrected*.

*eMCI, early mild cognitive impairment; lMCI, late mild cognitive impairment; SCD, subjective cognitive decline; HC, healthy controls; MNI, Montreal Neurological Institute; L, left hemisphere; R, right hemisphere; B, bilateral hemisphere*.

### Behavioral Significance of the Abnormal Functional Connectivity

**Figure 2:**
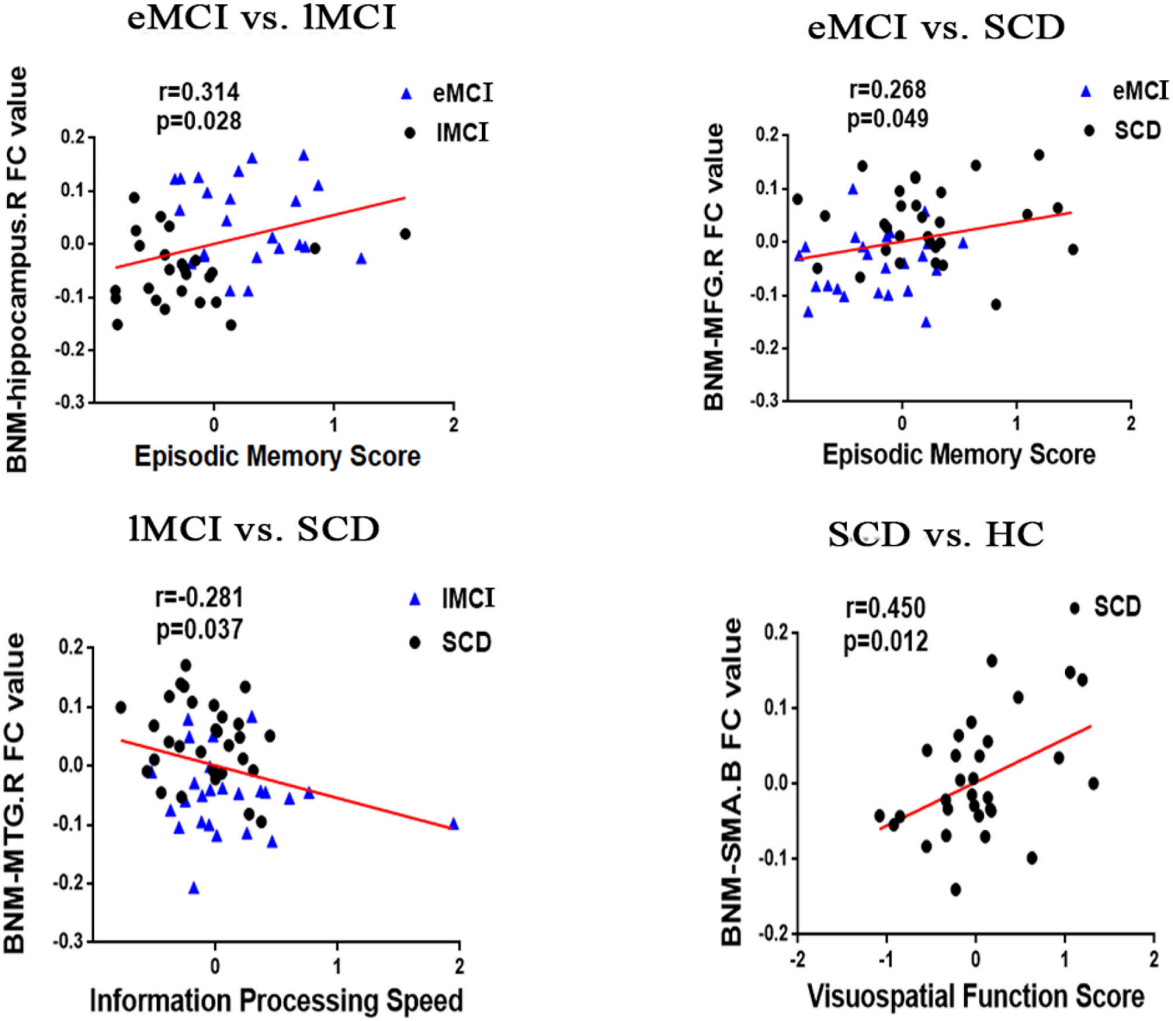
Significant associations between altered BNM-FC and cognitive function including episodic memory, executive function, and information processing speed (Bonferroni corrected, *p* < 0.05). Age, gender, and years of education were used as covariates for all these results. eMCI, early mild cognitive impairment; lMCI, late mild cognitive impairment; SCD, subjective cognitive decline; HC, healthy controls; SMA, supplementary motor area; SFG, superior frontal gyrus; MFG, middle frontal gyrus; L, left hemisphere; R, right hemisphere; B, bilateral hemisphere.

In the groups consisting of eMCI and lMCI, the analysis showed that the altered FC between the BNM and the right hippocampus is positively correlated with EM (*r* = 0.314, *p* = 0.028). Altered FC between the BNM and the right MFG was positively correlated with EM (*r* = 0.268, *p* = 0.049). Compared with SCD subjects, BNM-FC in the right MTG of lMCI subjects was negatively correlated with IPS (*r* = −0.281, *p* = 0.037). Compared with HC, BNM-FC in bilateral SMA of SCD subjects was positively correlated with VF (*r* = 0.450, *p* = 0.012). Age, gender, and years of education were used as covariates for all these results. There was no statistically significant correlation (Bonferroni corrected, *p* < 0.05) between the cognition domains and the remaining areas (Figure 2).

### Classification Results

**Figure 3:**
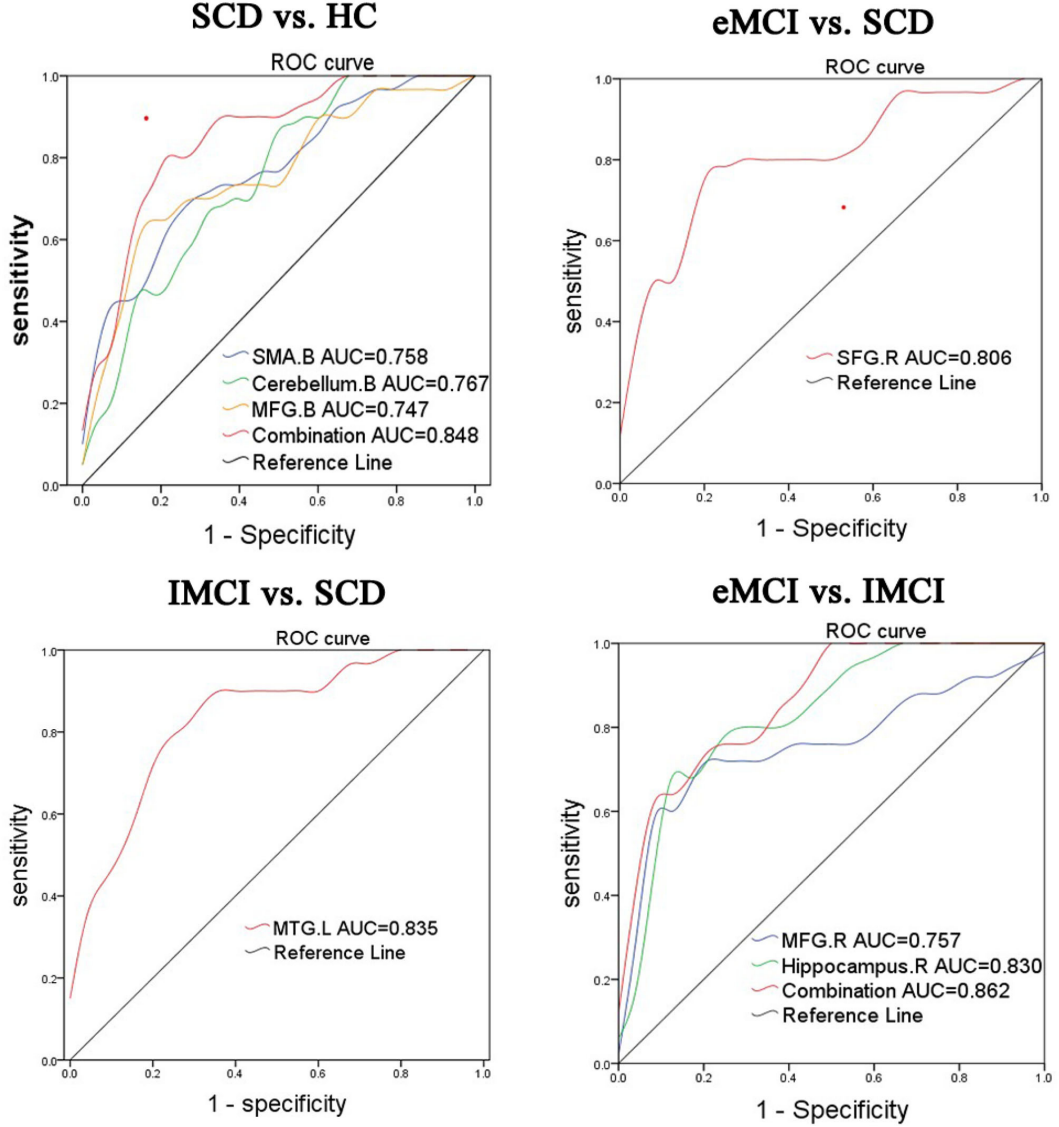
Receiver operating characteristic (ROC) curves of different groups were identified in specific brain areas. eMCI, early mild cognitive impairment; lMCI, late mild cognitive impairment; SCD, subjective cognitive decline; HC, healthy controls; SMA, supplementary motor area; SFG, superior frontal gyrus; MFG, middle frontal gyrus; MTG, middle temporal gyrus; L, left hemisphere; R, right hemisphere; B, bilateral hemisphere.

To further illustrate the classification performance of altered BNM-FC and their combination, we plot their ROC curves and presented their AUC values, respectively (Figure 3). AUC values for BNM-FC in the MFG, SMA, and cerebellum and their combination were 0.744, 0.758, 0.767, and 0.848. AUC values for BNM-FC in the MTG were 0.835. AUC values for BNM-FC in the SFG were 0.806. AUC values for BNM-FC in the MFG and hippocampus and their combination were 0.757, 0.830, and 0.862.

## Discussion

In this present study, we first used a seed-based method to examine the BNM-FC for the early AD spectrum (SCD, eMCI, and lMCI) and explored the relationship between altered BNM-FC and cognitive function. The main results were summarized below: (1) abnormal BNM-FC in the frontal lobe, occipital lobe, and cerebellum was indicated in the AD disease spectrum (SCD, eMCI, and lMCI) relative to HC, and there was abnormal BNM-FC in the frontal lobe and temporal lobe between pairwise comparisons in the disease group. (2) From HC to SCD, and then to eMCI, the BNM-FC in the ECN was damaged; from SCD to eMCI, and then to lMCI, the BNM-FC in the DMN was damaged. (3) These abnormal BNM-FCs were confirmed to be associated with different cognitive domains impairment. (4) Furthermore, the ROC analyses of abnormal BNM-FC could accurately determine the classification ability of differentiating SCD from HC, SCD from eMCI, SCD from lMCI, and eMCI from lMCI. The findings supported cholinergic dysfunction as an important etiological mechanism of the early disease spectrum of AD.

### The Pattern of Abnormal BNM-FC in the AD Disease Spectrum Relative to HC

As a new concept in the field of dementia, SCD is a transitional stage between healthy state and MCI, with a high risk of becoming a pathological state (Hill et al., 2017). In the absence of objective clinical evidence to support cognitive impairment, SCD appears to be indistinguishable from normal aging (Rabin et al., 2017). However, some potential alterations did exist in patients with SCD in functional imaging (Eliassen et al., 2017). Compared with the HC group, the SCD group showed increased BNM-FC in bilateral SMA and decreased BNM-FC in bilateral MFG and cerebellum. The MFG region, which is a part of the dorsal lateral prefrontal cortex, is in charge of working memory and executive cognitive functions (Liu G. et al., 2020). Self-perceived abnormalities in the performance of daily life were the most common complaints, which may be associated with the damage of the MFG (Vega et al., 2016). Damage to the cerebellum has been a neglected point in the AD disease spectrum (Qi et al., 2019). Cerebro-cerebellar loops have been confirmed to have a great influence on cognition (Sierra et al., 2016). The decline of BNM-FC in these two brain regions indicated that brain function had changed in SCD patients, but the cognitive impairment was not obvious. The area under the curve of the MFG was 0.744, the area under the curve of the cerebellum was 0.767, the area under the curve of the SMA was 0.758, and the area under the curve of their combined value was 0.848. From these data, it can be known that the BNM-FCs of these three brain regions are able to distinguish between SCD and HC. As a result, more attention must be paid to the specific brain regions of SCD patients with close follow-up. As for the increase of compensatory motor area, it can be understood as a compensatory situation after the loss of executive function and cerebellar balance function.

Compared with the HC group, increased BNM-FC in bilateral PCUN was indicated in the eMCI group, and increased BNM-FC in the right LING was shown in the lMCI group. Meanwhile, differences between the two disease groups were also significant. As the precursor stage of AD, the eMCI group indicated in the results of this paper that bilateral PCUN was the first to be affected. As part of the posterior parietal cortex, PCUN is involved in episodic memory, visual space, self-related information processing, metacognition, consciousness, and other processes (Koch et al., 2018). The clinical symptoms of eMCI are mild and have a little impact on life (Edmonds et al., 2019). However, previous articles have confirmed that PCUN may gradually alter as early as about 10–20 years before the onset of cognitive impairment, which is not difficult to understand, because AD is a disease related to genes and inheritance (Bateman et al., 2012; Riedel et al., 2016). LING, which is the core of visual network, is responsible for processing visual memory and logical analysis (Lenoir and Siéroff, 2019). As we know, lMCI is the aggravating stage of eMCI. In addition to simple memory damage, visual processing damage is more prominent, which may lead to misperception and miscommunication with the outside world (Liu et al., 2017). Overall, patients with eMCI have early impairments in episodic memory and information processing, while patients with lMCI may have more severe impairments in visual processing.

### The Pattern of Abnormal BNM-FC Between Pairs of Comparison Groups in the AD Disease Spectrum

Interestingly, in the process of disease transformation (from SCD to eMCI), decreased BNM-FC in the SFG was observed. According to the statistics of studies, most patients diagnosed with eMCI mainly complain of emotional and personality changes in addition to memory decline, which is consistent with the alteration of the SFG. That is to say, when the illness state is reached, a change in personality follows (Liu Y. et al., 2020). The evidence supporting our inference is also the ROC curve, with an area under the curve of 0.806, which indicates that the SFG has certain accuracy in distinguishing SCD from eMCI. In addition, MTG attenuation was observed in the transition from SCD to lMCI. Notably, MTG is a brain region with complex and diverse functions in memory processing (Gao et al., 2020). The results suggested that the biggest difference between subhealth and advanced development is in memory. Memory impairment is persistent and increases with the course of the disease until it progresses to AD, an irreversible mental impairment (Gao et al., 2020). The area of the ROC curve was 0.835, showing that the MTG is reliable in distinguishing SCD from lMCI.

In terms of the differentiation of the two subtypes of aMCI, we found that with the progression of aMCI (from eMCI to lMCI), decreased BNM-FC in the hippocampus and MFG could be detected. Located in the medial temporal lobe, the hippocampus plays a role in short-term memory, long-term memory, and spatial orientation (Chen et al., 2015). LMCI is the closest prodrome to AD, and hippocampal destruction can be predicted to undoubtedly cause disturbance to daily life, with a higher probability of turning into irreversible AD (Lisman et al., 2017). Previous evidence indicated that during the transformation from eMCI to lMCI, the hippocampal volume changed significantly, which is consistent with our findings (Hong et al., 2015). There has been numerous research on the hippocampus, but the results of our paper indicated that the hippocampus is a target of attention in terms of MCI conversion and progression. The area under the curve of the MFG was 83.0%, the area under the curve of the hippocampus was 73.7%, and the area under the curve of their combined value was 86.2%. Both the functional connectivity values of individual brain regions and the comprehensive functional connectivity values show specificity and sensitivity in distinguishing between eMCI and lMCI.

### Damaged Networks During Disease Progression

In the pairwise comparison, we found abnormal BNM-FC and further summarized some rules. The SCD group showed decreased BNM-FC in the MFG relative to the HC group, the eMCI group showed decreased BNM-FC in the MFG relative to the SCD group, and the lMCI group showed decreased BNM-FC in the MFG relative to the eMCI group. This result indicated that BNM-FCs in the frontal gyrus are continuously weakened during the transformation of a healthy state into eMCI. The SFG and MFG belong to the executive control network (ECN), which is involved in the regulation of cognition and behavior (Qi et al., 2010). Therefore, we can speculate that BNM-FCs in the ECN are disconnected in the qualitative change process from HCs to SCD, then to eMCI and then to lMCI.

Meanwhile, the eMCI group showed decreased BNM-FC in the hippocampus relative to the SCD group, and the lMCI group showed decreased BNM-FC in the MTG relative to the SCD group. The MTG and hippocampus pertain to DMN, which is an important index to evaluate the level of consciousness of patients (Grieder et al., 2018). In Alzheimer's disease, the DMN is the first to be compromised by amyloid deposition caused by the course of the disease (Grieder et al., 2018). In conclusion, whether the transition is from SCD to eMCI or lMCI, there is persistent temporal lobe damage and connectivity disruption between the BNM and DMN.

### Correlation Between BNM-FC and Cognitive Domains

Meanwhile, our findings indicated that episodic memory is positively correlated with BNM-FC in the hippocampus and MFG. Information processing speed is negatively correlated with BNM-FC in the MTG. Visuospatial function is negatively correlated with BNM-FC in the SMA. These results suggested that the abnormal BNM-FCs we found were specific and highly correlated with cognitive domains. From this, we can speculate that these abnormal BNM-FCs can be used as specific imaging markers in the AD early spectrum and further illustrate that AD is a disconnection syndrome.

### Limitation

Although the results of our article have been of great value, there are still some limitations that deserve our attention. First, differences in age and education among the four groups are inevitable. In order to avoid interference caused by these factors, we used age, sex, and education level as covariables in the statistical analysis. Overall, our results are fairly reliable. Second, this paper based on a cross-sectional design is a study of a small sample size. However, high-quality patient data rarely interfered with the paper's results. Further patient recruitment is ongoing and follow-up is being synchronized. We will make every effort to avoid possible deviations and seek more precise results.

## Conclusion

The abnormal BNM-FC patterns can characterize the spectrum of early AD (SCD, eMCI, and lMCI) and are closely related to the cognitive domains. These new and reliable findings will provide a new perspective in identifying the early disease spectrum of AD and further strengthen the role of cholinergic theory in AD.

## Data Availability Statement

The datasets presented in this article are not readily available because none. Requests to access the datasets should be directed to 1074914057@qq.com.

## Ethics Statement

The studies involving human participants were reviewed and approved by the responsible Human Participants Ethics Committee of the Affiliated Brain Hospital of Nanjing Medical University. The patients/participants provided their written informed consent to participate in this study. Written informed consent was obtained from the individual(s) for the publication of any potentially identifiable images or data included in this article.

## Author Contributions

All authors listed have made a substantial, direct and intellectual contribution to the work, and approved it for publication.

## Conflict of Interest

The authors declare that the research was conducted in the absence of any commercial or financial relationships that could be construed as a potential conflict of interest. The reviewer Y-CC declared a shared affiliation, with no collaboration, with the authors to the handling editor at the time of the review.
