# Supplementary material for: Altered Functional Connectivity of the Basal Nucleus of Meynert in Subjective Cognitive Impairment, Early Mild Cognitive Impairment, and Late Mild Cognitive Impairment
Source: Front Aging Neurosci. 2021 Jun 25;13:671351. doi: 10.3389/fnagi.2021.671351 (PMC8267913; doi:10.3389/fnagi.2021.671351)
Supplement: Supplementary file 1 [file Data_Sheet_1.docx]

# Supporting Information

# SI Methods

### S.1 NBH-ADsnp database

### S.2 Neuropsychological assessments for the NBH-ADsnp database

### S.3 Image acquisition for the NBH-ADsnp database

### S.4 Image preprocessing

### S.5 Selection of seed-mask

**S.1 NBH-ADsnp database**

Data used in this study were obtained from the Nanjing Brain Hospital-Alzheimer’s Disease (AD) Spectrum Neuroimaging Project (NBH-ADsnp) database (in-home website: http://192.168.8.100) (Nanjing, China). NBH-ADsnp was derived from an AD Spectrum Neuroimaging Project that was launched in January 2018 by the Institute of Brain Functional Imaging, the Affiliated Brain Hospital of Nanjing Medical University (Nanjing, China). Prof. Jiu Chen, PhD, MD, from the Affiliated Brain Hospital of Nanjing Medical University, served as the principal investigator of NBH-ADsnp. NBH-ADsnp was initiated by Dr. Jiu Chen and Dr. Xiangrong Zhang and was named by Dr. Jiu Chen's research group (discussed by Chen Xue, Guanjie Hu, Wenwen Xu, Wan Liu, Wenzhang Qi, Siyu Wang, Jiani Xu, Shanshan Chen, and finally verified by Jiu Chen and Xiangrong Zhang). NBH-ADsnp is an observational study, which includes cross-sectional and longitudinal follow-up components. The goal of NBH-ADsnp was to identify early neuroimaging biomarkers of preclinical AD spectrum (Eliassen et al., 2017 and AD and AD), to predict disease progression of individuals within the preclinical AD spectrum, and to provide imaging-based targets for individualized intervention to prevent disease deterioration from preclinical stages to the eventually progressed AD. Initially, several hundreds of elderly individuals in NBH-ADsnp, who were all Han Chinese and right-handed, were recruited from hospitals and local communities by advertising and by means of broadcasting. This database used a standardized clinical evaluation protocol that included a medical history interview, neurologic examination, a battery of neurocognitive assessments, and a resting-state MRI scan (T1, T2, 3D T1, DTI, and BOLD) for all participants (healthy controls (HC), SCD, naMCI, aMCI, and AD). All subjects and their study partners completed the informed consent process, and study protocols were reviewed and approved by the responsible Human Participants Ethics Committee of the Affiliated Brain Hospital of Nanjing Medical University (No. 2018-KY010-01, No. 2020-KY010-02, and No. ChiCTR1900022287).

### S.2 Neuropsychological Assessments

Neuropsychological assessments were performed as described in our previous research papers (Chen et al., 2019; Chen, Duan, et al., 2016; Chen et al., 2015; Chen et al., 2020; Xue et al., 2019). All subjects had a standardized clinical interview and underwent comprehensive neuropsychological assessments by 3 neuropsychologists (Dr. Xue, Qi, and Liu). The evaluation included the Mini Mental State Examination (MMSE), Montreal Cognitive Assessment (MoCA), Mattis Dementia Rating Scale (MDRS), Auditory Verbal Learning Test - immediate recall (AVLT-IR), Auditory Verbal Learning Test -5-min delayed recall (AVLT-5-min-DR), Auditory Verbal Learning Test-20-min delayed recall (AVLT-20-min-DR), Logical Memory Test-immediate recall (LMT-IR), Logical Memory Test-20-min delayed recall (LMT-20-min-DR), Rey-Osterrieth Complex Figure Test -20-min delayed recall (ROCFT-20min-DR), Clock Drawing Test (CDT), Rey-Osterrieth Complex Figure Test (ROCFT), Verbal Fluency Test (VFT), Digit Span Test (DST), Digital Symbol Substitution Test (DSST), Trail-Making Tests A and B (TMT-A and B), Stroop Color and Word Test A, B, and C, and Semantic Similarity (Similarity) test. AVLT-20-min DR, LMT-20-min DR, and CFT-20-min DR constitute the episodic memory (EM) score. DSST, TMT-A, Stroop A, and Stroop B make up the information processing speed (ISP) score. CFT and CDT form visuospatial function (VF) score. VFT, DST-backward, TMT-B, Stroop C, and Semantic Similarity constitute executive function (EF) score. These tests were used to evaluate general cognitive function, episodic memory, information processing speed, executive function, and visuo-spatial function.

**S.3 MRI Data Acquisition**

### The NBH-ADsnp data acquisition process was also recorded in our former articles(Chen et al., 2019; Chen, Shu, et al., 2016; Xue et al., 2019). The acquisition of magnetic resonance imaging (MRI) data is all obtained through using a 3.0 Tesla Verio Siemens scanner with an 8-channel head-coil in the Affiliated Brain Hospital of Nanjing Medical University. The subjects were asked to close their eyes, stop thinking and remain still to ensure accurate resting state images. 240 volumes were included in the the echo-planar imaging (EPI) sequence. The parameters were: repetition time (TR) = 2,000 ms, echo time (TE) = 30 ms, number of slices = 36, thickness = 4.0 mm, gap = 0 mm, matrix = 64 × 64, flip angle (FA) = 90°, field of view (FOV) = 220 mm × 220 mm, acquisition bandwidth = 100 kHz, and voxel size = 3.4 × 3.4 × 4 mm^3^. The imaging took approximately 8 minutes.

High-resolution T1-weighted images were acquired by 3D magnetization-prepared rapid gradient-echo (MPRAGE) sequence, whose parameters were as follows: TR = 1,900 ms, TE = 2.48 ms, inversion time (TI) = 900 ms, number of slices = 176, thickness = 1.0 mm, gap = 0.5 mm, matrix = 256 × 256, FA = 9°, FOV = 256 mm × 256 mm, and voxel size = 1 × 1 × 1 mm^3^. The imaging took approximately 4.26 minutes.

Routine axial T2-weighted images were acquired to rule out subjects with major changes in white matter (WM), cerebral infarction or other lesions using flair sequence as follows: TR = 8400 ms, TE = 94 ms, FA= 150°, acquisition matrix = 256×256, FOV = 230×230 mm, thickness = 5.0 mm, gap = 0 mm, and number of slices = 20. The imaging processtook approximately 2.50 minutes to complete.

**S.4 Image preprocessing**

The image processing procedures were performed as previously described by Yan et al(Yan, Craddock, Zuo, Zang, & Milham, 2013). All fMRI data were preprocessed using MATLAB 2013b (http://www.mathworks.com/products/matlab/) and DPABI image processing software(Yan, Wang, Zuo, & Zang, 2016). To maintain the stability of the MRI signal, the first 10 volumes were removed. Slice timing and motion effects were first corrected. Corrections were performed for the intra-volume acquisition time differences among slices and inter-volume motion effects during the scan(Power, Barnes, Snyder, Schlaggar, & Petersen, 2012; Van Dijk, Sabuncu, & Buckner, 2012). Functional and structural images were co-registered. Structural images were then normalized and segmented into gray matter (Note: gray matter was used as a covariate in statistical comparison between groups), white matter and CSF partitions using the DARTEL technique(Ashburner & Friston, 2009). A Friston 24-parameter model was used to regress out head motion effects from the realigned data(Friston, Williams, Howard, Frackowiak, & Turner, 1996). CSF, white matter, and the global signals as well as the linear trend were also regressed as nuisance covariates(Brady et al., 2019). After realigning, slice-timing correction, and co-registration, framewise displacement (FD) was calculated for all resting-state volumes(Power et al., 2012). All volumes with a FD greater than 0.2 mm were regressed out as nuisance covariates(Brady et al., 2019). The fMRI data were spatially normalized to a standard EPI template and were resampled to 3×3×3 mm^3^ voxels(Chen, Shu, et al., 2016). Finally, functional images were spatially smoothed with a Gaussian kernel of 6×6×6 mm (full width at half maximum, FWHM(Chen, Shu, et al., 2016)). Temporal band-pass filtering (0.01–0.1 Hz) was applied to reduce the effect of low-frequency drifts and high-frequency physiological noise. Voxels within a group GM mask, created by DARTEL, were used for further analyses(Yan et al., 2013; Zhou et al., 2019).

**S.5 Selection of seed mask**

The seed BNM was, as defined by Li et al.(C. S. Li et al., 2014), based on a stereotaxic probabilistic maps of the basal forebrain, that contains the magnocellular cholinergic corticopetal projection neurons(Zaborszky et al., 2008). In the latter study, Hui Li et al.(H. Li et al., 2017) made 10 human postmortem brains into histological serial sections and stained by silver. The positions and the extent of each part of the basal forebrain were microscopically delineated, 3D reconstructed and warped to the reference space of the MNI brain. Magnocellular cell groups in the subcommissural-sublenticular region of the basal forebrain were the definition of BNM (de Lacalle, Iraizoz, & Ma Gonzalo, 1991; Vogels et al., 1990).

**Reference**

Ashburner, J., & Friston, K. J. (2009). Computing average shaped tissue probability templates. *Neuroimage, 45*(2), 333-341. doi:10.1016/j.neuroimage.2008.12.008

Brady, R. O., Jr., Gonsalvez, I., Lee, I., Öngür, D., Seidman, L. J., Schmahmann, J. D., . . . Halko, M. A. (2019). Cerebellar-Prefrontal Network Connectivity and Negative Symptoms in Schizophrenia. *Am J Psychiatry, 176*(7), 512-520. doi:10.1176/appi.ajp.2018.18040429

Chen, J., Chen, G., Shu, H., Chen, G., Ward, B. D., Wang, Z., . . . Zhang, Z. (2019). Predicting progression from mild cognitive impairment to Alzheimer's disease on an individual subject basis by applying the CARE index across different independent cohorts. *Aging (Albany NY), 11*(8), 2185-2201. doi:10.18632/aging.101883

Chen, J., Duan, X., Shu, H., Wang, Z., Long, Z., Liu, D., . . . Zhang, Z. (2016). Differential contributions of subregions of medial temporal lobe to memory system in amnestic mild cognitive impairment: insights from fMRI study. *Sci Rep, 6*, 26148. doi:10.1038/srep26148

Chen, J., Shu, H., Wang, Z., Liu, D., Shi, Y., Zhang, X., & Zhang, Z. (2015). The interaction of APOE genotype by age in amnestic mild cognitive impairment: a voxel-based morphometric study. *J Alzheimers Dis, 43*(2), 657-668. doi:10.3233/jad-141677

Chen, J., Shu, H., Wang, Z., Zhan, Y., Liu, D., Liao, W., . . . Zhang, Z. (2016). Convergent and divergent intranetwork and internetwork connectivity patterns in patients with remitted late-life depression and amnestic mild cognitive impairment. *Cortex, 83*, 194-211. doi:10.1016/j.cortex.2016.08.001

Chen, J., Shu, H., Wang, Z., Zhan, Y., Liu, D., Liu, Y., & Zhang, Z. (2020). Intrinsic connectivity identifies the sensory-motor network as a main cross-network between remitted late-life depression- and amnestic mild cognitive impairment-targeted networks. *Brain Imaging Behav, 14*(4), 1130-1142. doi:10.1007/s11682-019-00098-4

de Lacalle, S., Iraizoz, I., & Ma Gonzalo, L. (1991). Differential changes in cell size and number in topographic subdivisions of human basal nucleus in normal aging. *Neuroscience, 43*(2-3), 445-456. doi:10.1016/0306-4522(91)90307-a

Eliassen, C. F., Reinvang, I., Selnes, P., Grambaite, R., Fladby, T., & Hessen, E. (2017). Biomarkers in subtypes of mild cognitive impairment and subjective cognitive decline. *Brain Behav, 7*(9), e00776. doi:10.1002/brb3.776

Friston, K. J., Williams, S., Howard, R., Frackowiak, R. S., & Turner, R. (1996). Movement-related effects in fMRI time-series. *Magn Reson Med, 35*(3), 346-355. doi:10.1002/mrm.1910350312

Li, C. S., Ide, J. S., Zhang, S., Hu, S., Chao, H. H., & Zaborszky, L. (2014). Resting state functional connectivity of the basal nucleus of Meynert in humans: in comparison to the ventral striatum and the effects of age. *Neuroimage, 97*, 321-332. doi:10.1016/j.neuroimage.2014.04.019

Li, H., Jia, X., Qi, Z., Fan, X., Ma, T., Ni, H., . . . Li, K. (2017). Altered Functional Connectivity of the Basal Nucleus of Meynert in Mild Cognitive Impairment: A Resting-State fMRI Study. *Front Aging Neurosci, 9*, 127. doi:10.3389/fnagi.2017.00127

Power, J. D., Barnes, K. A., Snyder, A. Z., Schlaggar, B. L., & Petersen, S. E. (2012). Spurious but systematic correlations in functional connectivity MRI networks arise from subject motion. *Neuroimage, 59*(3), 2142-2154. doi:10.1016/j.neuroimage.2011.10.018

Van Dijk, K. R., Sabuncu, M. R., & Buckner, R. L. (2012). The influence of head motion on intrinsic functional connectivity MRI. *Neuroimage, 59*(1), 431-438. doi:10.1016/j.neuroimage.2011.07.044

Vogels, O. J., Broere, C. A., ter Laak, H. J., ten Donkelaar, H. J., Nieuwenhuys, R., & Schulte, B. P. (1990). Cell loss and shrinkage in the nucleus basalis Meynert complex in Alzheimer's disease. *Neurobiol Aging, 11*(1), 3-13. doi:10.1016/0197-4580(90)90056-6

Xue, C., Yuan, B., Yue, Y., Xu, J., Wang, S., Wu, M., . . . Chen, J. (2019). Distinct Disruptive Patterns of Default Mode Subnetwork Connectivity Across the Spectrum of Preclinical Alzheimer's Disease. *Front Aging Neurosci, 11*, 307. doi:10.3389/fnagi.2019.00307

Yan, C. G., Craddock, R. C., Zuo, X. N., Zang, Y. F., & Milham, M. P. (2013). Standardizing the intrinsic brain: towards robust measurement of inter-individual variation in 1000 functional connectomes. *Neuroimage, 80*, 246-262. doi:10.1016/j.neuroimage.2013.04.081

Yan, C. G., Wang, X. D., Zuo, X. N., & Zang, Y. F. (2016). DPABI: Data Processing & Analysis for (Resting-State) Brain Imaging. *Neuroinformatics, 14*(3), 339-351. doi:10.1007/s12021-016-9299-4

Zaborszky, L., Hoemke, L., Mohlberg, H., Schleicher, A., Amunts, K., & Zilles, K. (2008). Stereotaxic probabilistic maps of the magnocellular cell groups in human basal forebrain. *Neuroimage, 42*(3), 1127-1141. doi:10.1016/j.neuroimage.2008.05.055

Zhou, C., Yu, M., Tang, X., Wang, X., Zhang, X., Zhang, X., & Chen, J. (2019). Convergent and divergent altered patterns of default mode network in deficit and non-deficit schizophrenia. *Prog Neuropsychopharmacol Biol Psychiatry, 89*, 427-434. doi:10.1016/j.pnpbp.2018.10.012
